# Safety and Toxicology Profile of TT-6-AmHap Heroin Conjugate Vaccine

**DOI:** 10.3390/vaccines13080792

**Published:** 2025-07-26

**Authors:** Essie Komla, Erwin G. Abucayon, C. Steven Godin, Agnieszka Sulima, Arthur E. Jacobson, Kenner C. Rice, Gary R. Matyas

**Affiliations:** 1 Laboratory of Adjuvant and Antigen Research, US Military HIV Research Program, Center for Military Infectious Diseases Research, Walter Reed Army Institute of Research, 503 Robert Grant Avenue, Silver Spring, MD 20910, USA; ekomla@hivresearch.org (E.K.); erwin.abucayon@merck.com (E.G.A.); 2 Henry M. Jackson Foundation for the Advancement of Military Medicine, 6720A Rockledge Drive, Bethesda, MD 20817, USA; 3 Inotiv, 13 Firstfield Rd., Suite 110, Gaithersburg, MD 20878, USA; c.steven.godin@inotiv.com; 4 Drug Design and Synthesis Section, Molecular Targets and Medications Discovery Branch, Intramural Research Program, National Institute on Drug Abuse and the National Institute on Alcohol Abuse and Alcoholism, National Institutes of Health, Department of Health and Human Services, 9800 Medical Center Drive, Bethesda, MD 20892, USA; agnieszka.sulima@nih.gov (A.S.); arthurj@nida.nih.gov (A.E.J.); kennerr@mail.nih.gov (K.C.R.)

**Keywords:** heroin vaccine, opioid, adjuvants, vaccine efficacy, conjugate vaccine, toxicity, safety, opioid use disorder, rabbits

## Abstract

**Background/Objectives**: Opioid use disorder (OUD) remains a severe health problem globally, resulting in substantial social and economic challenges. While existing medications for managing OUD are proven to be effective, they also present certain challenges. A vaccine offers a promising therapeutic strategy to combat OUD and potentially reduce the risk of overdose death. The TT-6-AmHap heroin conjugate vaccine has effectively reduced heroin-induced pharmacological effects in behavioral assays as well as demonstrated the induction of high titer and high affinity antibody responses in mice and rats. In this GLP study conducted in rabbits, the potential local and systemic toxicity of the TT-6-AmHap heroin vaccine in combination with or without adjuvants ALF43 and Alhydrogel^®^ (ALFA) was investigated. **Methods**: Male and female New Zealand White rabbits were administered with vaccines or a saline control intramuscularly at two-week intervals over a 57-day study period. The presence, persistence or reversibility of any toxic effects of the vaccine was determined over a four-week recovery period. **Results**: Administration of TT-6-AmHap with or without the adjuvants induced high antibody-specific IgG in treatment groups compared to the controls. The study found no TT-6-AmHap-related effects on mortality, physical examinations, dermal Draize observations, body weights, body weight changes, food consumption, ophthalmology, clinical pathology (hematology, coagulation, clinical chemistry, and urinalysis), macroscopic pathology, or organ weights. **Conclusions**: Under the conditions of this study, these results demonstrate that the TT-6-AmHap vaccine with or without adjuvants was well tolerated, immunogenic, and the effects were not considered adverse in both male and female rabbits.

## 1. Introduction

Opioid use disorder (OUD) continues to be a global public health crisis. In the United States alone, the reported opioid overdose deaths as of December 2024 were approximately 55,000 [1]. Although current FDA-approved medications for OUD treatment have shown their effectiveness, they are insufficient in addressing the ongoing OUD and overdose crisis [2,3]. This highlights the need for innovative therapies to address the evolving landscape of OUD. A promising approach to treating OUD is the development of anti-opioid vaccines, which stimulate the production of opioid hapten-specific antibodies [4,5]. Drugs of abuse are typically small molecules with low molecular weights and are not immunogenic. To elicit immune response, they require conjugations to larger immunogenic carrier proteins, which are detected by the immune system [6,7,8]. Commonly used protein carriers for conjugate opioid vaccines include tetanus toxoid (TT), diphtheria toxoid (DT), and keyhole limpet hemocyanin (KLH) [9,10,11]. To enhance the efficacy of opioid vaccines, incorporating adjuvants is necessary [12], as they facilitate the production of high-affinity antibodies that bind to opioids, preventing them from crossing the blood–brain barrier, and reducing their physiological effects.

The TT-6-AmHap heroin vaccine developed in our laboratories uses tetanus toxoid as a carrier and Army Liposome Formulation (ALF43) adsorbed to aluminum hydroxide (Alhydrogel^®^) (ALFA) as adjuvants [13,14]. TT has an acceptable clinical safety profile and is used as the antigen for the licensed tetanus vaccine and in several licensed vaccines [15,16,17,18]. TT-6-AmHap has consistently shown high vaccine efficacy in mice and rats in behavioral assays [14,19]. Moreover, preexisting immunity to TT from DTaP vaccination has no effects on the efficacy of the vaccine [9].

Although numerous pre-clinical studies in animal models have demonstrated the effectiveness of opioid vaccines in reducing opioid-induced antinociception, hyperlocomotion, respiratory depression, bradycardia, and overdose [14,19,20,21,22,23,24,25,26], the complete toxicity profile of these vaccines remains largely unexplored. To date, there is only one published report on the safety and toxicology profile of an opioid vaccine targeting oxycodone use disorder [27]. As described, the Oxy(Gly)4-sKLH oxycodone vaccine was safe, immunogenic, and well tolerated in rats [27].

In this paper, we describe the results of a GLP pharmacology-toxicology study in rabbits to determine the potential local and systemic toxicity of the TT-6-AmHap heroin vaccine in combination with or without adjuvants ALF43 and Alhydrogel^®^ (ALFA). The Parameters evaluated included but not limited to physical examinations, clinical pathology, and vaccine-induced antibody response. The results showed no TT-6-AmHap-related effects on the parameters tested. Notably, the TT-6-AmHap vaccine with or without adjuvants was well tolerated, immunogenic, and none of the effects of the vaccine were deemed detrimental.

## 2. Materials and Methods

### 2.1. Ethics

This pharmacology-toxicology study was performed by Inotiv and conducted in compliance with current U.S. Food and Drug Administration (FDA) Good Laboratory Practice (GLP) Regulations for Non-clinical Laboratory Studies (21 CFR Part 58). The Institutional Animal Care and Use Committee (IACUC) of Inotiv approved the study protocol and found it to be in accordance with provisions of the United States Department of Agriculture (USDA) Animal Welfare Act, the Public Health Service (PHS) Policy on Humane Care and Use of Laboratory Animals, and the US Interagency Research Animal Committee Principles for the Utilization and Care of Research Animals.

### 2.2. Animals

New Zealand White rabbits (specific pathogen-free—SPF) were purchased from Charles River Breeding Labs (Saint Constant, Canada). Rabbits were selected based on regulatory recommendations as standard species for use in toxicology studies of vaccine candidates and assessing the safety of these candidates. Animals were individually housed in multiple rooms in polycarbonate cages mounted on stainless steel racks. Animals were identified with a microchip and a cage card containing pertinent animal and study information. Animals were provided unrestricted access to Certified Global Teklad Laboratory Diet 2030 and filtered water via an automated watering system, supplemented with filtered water bottles when necessary. Animals were acclimated to laboratory conditions for 16 days and released from acclimation by a staff veterinarian. During acclimation, animals received one cup of food per day. Animals were subsequently provided feed ad libitum, unless otherwise noted. The feed was analyzed by the manufacturer for concentrations of specified heavy metals, aflatoxin, chlorinated hydrocarbons, and organophosphates. Water was provided ad libitum and was routinely analyzed for contaminants and specific microbes. No contaminants were known to be present in the feed or water at levels that might have interfered with achieving the objectives of the study. At the initiation of dosing, male rabbits were 15 weeks old with body weights ranging from 2648 to 3381 g and female rabbits were 15 weeks old with body weights ranging from 3238 to 3749 g. A standard 12 h light/dark was used. Environmental conditions were maintained at a minimum of 10 air changes per hour, with interruptions permitted only for study-related procedures.

### 2.3. Vaccine Formulations

All vaccine products were manufactured on cGMP conditions.

TT-6-AmHap

6-AmHap thio acetate was manufactured by Avista Pharma Solution Inc., Durham, NC. The synthesis was an 8-step process starting from hydromorphone. The resulting 6-AmHap was placed on a 9-month stability program and was stable for that period. Tetanus toxoid (TT) was purchased from MassBiologics, Boston, MA, USA. SM-(PEG)2 was from Thermo Fisher Scientific, Waltham, MA, USA. It was not manufactured under cGMP, but was extensively tested to ensure purity prior to use for the conjugation. The 6-AmHap was conjugated to TT using the SM-(PEG)2 linker under cGMP at the WRAIR Pilot Bioproduction Facility (PBF) using a scaled-up method originally described [14]. Briefly, the TT was buffer exchanged to 100 mM sodium phosphate, 150 mM sodium chloride, pH 7.2, using tangential flow filtration (TFF). The SM-(PEG)2 was dissolved in dimethyl sulfoxide (DMSO) and added to the TT. Following a 2 h incubation at room temperature, the excess SM-(PEG)2 was removed by TFF and the buffer was exchanged to 50 mM 4-(2-hydroxyethyl)-1-piperazineethanesulfonic acid (HEPES), pH 7.2. 6-AmHap thio acetate was dissolved in DMSO and added to the TT-linker. The reaction was stirred for 16 h at 4 °C. The buffer was exchanged with PBS, pH 7.4 and the TT-6-AmHap was concentrated by TFF. The TT-6-AmHap was vialed, stored at 4 °C and placed on a stability plan.

Alhydrogel^®^

Alhydrogel-2% was manufactured on cGMP by Brenntag Biosector, Frederikssund, Denmark. The Alhydrogel was diluted with 1.18% sodium chloride solution to yield 2400 µg/mL aluminum in 0.9% sodium chloride. The diluted Alhydrogel^®^ was vialed, stored at 4 °C and placed on a stability plan.

ALF43

Bulk ALF43 was manufactured by Avanti Research, Alabaster, AL. 1,2-dimyristoyl-*sn*-glycero-3-phosphocholine (DMPC) and cholesterol were solubilized in chloroform and 1,2-dimyristoyl-*sn*-glycero-3-phosphoglycerol (DMPG) and synthetic monophosphoryl lipid A (3D-PHAD^®^) were solubilized in chloroform:methanol 9:1 (*v/v*). The lipids were combined in a molar ratio of 9:7.5:1:1.14 and the solvent was removed by rotary evaporation [28]. The dried lipid cake was suspended in water and liposomes were reduced in size to 30–150 nm using a high-pressure emulsifier (Avestin, Inc., Ottawa, OT, Canada). The liposomes were passed through a polyethersulfone filter and filled into a TepoFlex^®^ biocontainer (Missner Filtration Products, Camarillo, CA, USA), which was shipped to the WRAIR PBF. The liposomes were removed from the bag and filtered through a polyethersulfone 0.22 µm filter (MilliporeSigma, Burlington, MA, USA). They were transfer to 5 mL glass vials and lyophilized [29]. The lyophilized ALF43 was stored a −20 °C and placed on a stability plan.

### 2.4. Dose Formulations

This study tested a vaccine formulation consisting of Current Good Manufacturing Practices (cGMP) manufactured TT-6-AmHap (lot 2066), ALF43 (lot 1960), Alhydrogel^®^ (lot 1968) from Pilot Bioproduction Facility (PBF) at Walter Reed Army Institute of Research (WRAIR), and 0.9% Sodium Chloride (saline) from Baxter International, Inc. (lot Y374385). The vaccine contained tetanus toxoid (TT) conjugated to 6-AmHap heroin hapten (TT-6-AmHap) with or without adjuvants. As determined by Matrix-Assisted Laser Desorption/Ionization (MALDI) with Time-of-Flight (TOF) mass spectroscopy, the approximate number of haptens conjugated to TT was 36 [14]. The adjuvants consisted of the Army Liposome Formulation containing 43% cholesterol (ALF43) adsorbed to aluminum hydroxide (Alhydrogel^®^), together known as ALFA. Group 1 was the vehicle/control saline group. Group 2 (TT-6-AmHap) formulation was prepared by mixing the required volumes of TT-6-AmHap and saline. The formulations were mixed by inversion 3–5 times and left to sit at room temperature (21 ± 5 °C) for at least 10 min. Group 3 (TT-6-AmHap with ALFA) formulation was prepared by mixing the required volumes of ALF43 and saline. Formulations were vortexed at 250 Revolutions Per Minute (RPM) for at least two minutes and left to sit at room temperature (21 ± 5 °C) for 10 min. Separately, the required volumes of TT-6-AmHap and Alhydrogel^®^ were mixed via inversion 3–5 times and left to sit at room temperature (21 ± 5 °C) for 10 min. 0.5 mL of the TT-6-AmHap and Alhydrogel^®^ mix was added to ALF43 formulation and mixed via inversion 3–5 times. Group 2 and 3 formulations were assigned a 4 h shelf life and stored on wet ice until used for dosing. See Table 1 for vaccine dose.

### 2.5. Study Design

Animals were randomized based upon pre-study body weights, physical examinations, and ophthalmic examinations. Study group assignment was conducted using computer-generated randomization, stratified by sex, to ensure that mean body weights did not differ significantly from the control group (*p* < 0.05). Upon completion of randomization, each animal was assigned a unique identifier for tracking and data collection purposes. The animals were assigned to groups (5 animals/sex/group/study phase) as shown in Table 1. All animals were dosed via intramuscular (IM) injection on Study Days (SD) 1, 15, 29, 43, and 57 at a volume of 0.5 mL/rabbit. Half of the animals were subjected to a full gross necropsy on SD 60 (terminal) and the other half were subjected to a full gross necropsy on SD 85 (recovery). Dosing sites alternated such that the right lateral thigh region was used on SD 1, 29, and 57 and the left lateral thigh region was used on SD 15 and 43. The first day of dosing was designated as SD 1 for each animal. All animals were dosed via IM as it is the intended route of administration in humans. The selected dose levels were established based on the proposed therapeutic dosing regimen, and the results of previous immunogenicity studies in mice and rats with no adverse clinical signs noted.

### 2.6. Animal Observations and Measurements

Animals were observed at least twice daily for mortality, moribundity, and general health. Physical examinations (skin and fur characteristics, eye and mucous membranes, respiratory, circulatory, autonomic and central nervous systems, somatomotor and behavior patterns, injection sites) were performed at least once before the first dose was administered (SD 1), then weekly thereafter, and prior to necropsy. Any unscheduled observations were also recorded. Dermal observations of injection sites were evaluated using the 5-point Draize scoring scale ranging from none to severe (0–4), with the 0 score being no swelling and normal color, to the score of 4 being pronounced swelling and dark red. The injection sites were evaluated prior to each dose, 1 h (±15 min) and 4 h (±30 min) following each dose, daily for the first 3 days following each dosing, and daily thereafter until no erythema or edema was noted. The dose site from the previous dosing(s) were scored at each subsequent time point. Ophthalmologic observations were conducted using indirect ophthalmoscopy and slit-lamp bio-microscopy (as needed); the eyes of each animal were examined following the administration of Tropicamide^®^ mydriatic solution. Ophthalmology examinations were conducted for all animals prior to randomization (SD 1), SD 58 and 84 for females; SD 59 and 85 for males, and for all surviving animals prior to scheduled necropsies. Body temperatures were collected by scanning the implanted microchip transponder. Body temperatures of all animals were recorded on the day prior to each dose, 3 h ± 15 min after each dose, 6 h ± 15 min after each dose, and 24 ± 1 h after each dose. If the temperature was greater than 40 °C, daily measurements were collected until the temperature fell below 40 °C.

### 2.7. Body Weights and Food Consumption

All animals were weighed at least once prior to initiation of dosing (SD 1), 24 and 48 ± 1 h after each dose, and weekly thereafter. Body weights were reported in grams. Food Consumption was recorded on a daily basis from arrival and the data was reported as grams/animal/day.

### 2.8. Clinical Pathology Sample Collection

Hematology blood samples (at least 1.0 mL), clinical chemistry (at least 2.0 mL), and coagulation (up to 1.8 mL) were collected from fasted animals via a medial auricular artery prior to the first dose, SD 2, 4, 56, 58, and prior to scheduled terminations (all surviving animals at each interval). Urine samples (Up to 12 mL) were collected from fasted animals prior to scheduled terminations (all surviving animals at each interval). Blood samples for hematology tests were collected in tubes containing potassium Ethylenediaminetetraacetic acid (EDTA) and tested on Siemens Advia 2120i hematology analyzer. Tubes containing sodium citrate were used for the coagulation tests and the Instrumentation Laboratory ACL Elite Pro coagulation analyzer was used to measure the coagulation tests. Serum separator tubes were used for clinical chemistry tests and Siemens Dimension Xpand chemistry analyzer was used to measure the serum concentrations of the samples. Urine samples were collected in a pan. The specimens were processed using reagent strips and ran on Siemens Clinitek Atlas analyzer or evaluated manually.

### 2.9. Postmortem Evaluations

Five animals of each sex from each dose group were humanely euthanized by intravenous injection of sodium pentobarbital and exsanguinated on SD 60. The remaining five surviving animals per sex from each dose group were observed for a post-dosing recovery period and euthanized on SD 85. The animals were necropsied after euthanasia. Macroscopic examinations were performed which included visual examination of the carcass for external abnormalities such as palpable masses. The abdominal, thoracic, and cranial cavities and their contents were examined for abnormalities. Protocol-specified tissues were preserved in 10% neutral buffered formalin (NBF) with the exception of the eyes (and associated ocular tissue), testes, and epididymis, which were preserved in modified Davidson’s fixative and subsequently transferred to 10% NBF. The preserved tissues were transferred to Inotiv, St. Louis, MO, USA, where they were embedded in paraffin, sectioned, stained with hematoxylin and eosin (H&E), and examined by a board-certified veterinary pathologist.

### 2.10. Evaluation of 6-AmHap-Specific Antibody Titer

Blood samples (at least 1.0 mL) were collected via a medial auricular artery into serum separator tubes. The samples were centrifuged at 1500 Relative Centrifugal Force (RCF) for 15 min at 5 ± 3 °C within 1 h of collection of each blood sample and the resultant serum was stored at −75 ± 15 °C until shipped on dry ice to WRAIR for analysis. Serum antibody analysis was performed via Enzyme-Linked Immunosorbent Assay (ELISA) under non-GLP conditions as previously described [9]. Briefly, 96-well microplates were coated with BSA-6-AmHap (1 μg/mL in 1× DPBS, pH 7.4). 100 μL of the coating solution was added to each plate well and incubated overnight at 4 °C. The following day, the coating solution was removed from plates. Blocking buffer (0.5% Milk, 0.1% Tween 20 in 1× Dulbecco’s Phosphate-Buffered Saline (DPBS), pH 7.4) was added to the plates and incubated at room temperature for 2 h. Positive control sera were previously prepared by pooling sera from Group 3 SD 71 mixed, aliquoted (100 μL per tube), and frozen at −80 °C. Both rabbit sera and positive control sera were diluted 1:400 in blocking buffer and added to the plates in triplicate. Serial dilutions of the sera were performed down the rows of each plate. Following a 2 h incubation at room temperature, the plates were washed with 0.1% Tween 20 in 1× DPBS. The secondary antibody (Goat anti-rabbit IgG-HRP) was diluted 1:5000 in blocking buffer and applied to the plates for a 1 h incubation at room temperature (21 ± 5 °C), followed by an additional wash. ABTS peroxidase substrate solution (100 μL/well) was then added and incubated for 1 h at room temperature (21 ± 5 °C). The reaction was terminated with 1% sodium dodecyl sulfate (SDS), and absorbance was measured immediately at 405 nm. The ELISA data was processed by averaging the absorbances at each dilution and calculating the standard deviation. The endpoint titers of samples were determined by averaging all “Blank or Background” absorbances and multiplied by 2. The endpoint titers of each sample were selected from the average value of each dilution and were closed to or above the “Blank × 2” value.

### 2.11. Quality Assurance

Quality assurance was conducted by Inotiv GLP Quality Assurance Unit (QAU) in accordance with the current U.S. FDA GLP Regulations for Non-clinical Studies (21 CFR Part 58), the protocol, and Inotiv Standard Operating Procedures (SOP).

### 2.12. Data Analysis

Data analysis, unless otherwise specified, was performed by Inotiv using Provantis™ Version 8 or greater (Instem LSS, Limited; Stone, UK). Quantitative data (body weights, body weight changes, body temperatures, food consumption, clinical pathology, and organ weights) generated from treatment groups were statistically compared to control group data using one-way Analysis of Variance (ANOVA), with males and females analyzed separately. Before ANOVA analysis, untransformed data were assessed for normality and homogeneity of variances using the Shapiro–Wilk and Levene’s tests, respectively. If either test yielded a *p*-value ≤ 0.01, data were log-transformed and re-evaluated. If assumptions were still not met, rank-transformed data were analyzed using the Kruskal–Wallis ANOVA. When assumptions were satisfied, ANOVA was conducted on either untransformed or log-transformed data as appropriate. Dunnett’s *t*-test was used for post hoc comparisons to identify significant differences from the control group. Statistical significance was set at *p* < 0.05 (two-tailed). For proportion or percentage data that violated parametric assumptions, an arcsine square root transformation was applied prior to analysis. 6-AmHap-specific antibody titers data were analyzed using two-way ANOVA (GraphPad Prism 9, v9.4.1; GraphPad Software, La Jolla, CA, USA) as described in the figure legend. A *post hoc* analysis was performed using Sidak’s multiple comparisons test to identify differences between Groups 2 and 3. Values represent the mean ± S.E.M. Level of significance in this study is indicated by a *p* value of *p* ≤ 0.05.

## 3. Results

### 3.1. Clinical Findings of Systemic Administration of TT-6-AmHap Vaccine

Treatment with TT-6-AmHap with or without adjuvants had no impact on mortality, with all animals surviving to the scheduled termination point. Physical examinations of the animals immunized with TT-6-AmHap with or without adjuvants were not significantly different from the controls. There were incidental findings of abrasions observed on the scrotum on Study Days (SD) 1 and on the nose on SD 85 in two Group 1 males and on SD 42 in one Group 2 male (Table 2). Abrasions on the nose and mouth were also observed on SD 85 in two Group 3 males (Table 2). These abrasions only occurred in male rabbits, in the control and treatment groups. Furthermore, there were incidental findings of discolored, brown urine observed on SD 73 and 78 in two Group 2 males and incidental findings of urine stain observed on SD 85 in one Group 2 male rabbit (Table 2). The observation only occurred in a small number of animals and did not persist. Additionally, there was no correlation with urinalysis findings (Table S1).

Immunization with TT-6-AmHap with or without adjuvants did not significantly affect dermal Draize observations. All animals but Group 1 female rabbits exhibited minimal edema and/or erythema at the site of injection at 1 h (hr), 4 h, and 24 h post second, third, and fourth dose administration. The edema and erythema were quickly resolved by the following day. The number of animals with normal Draize scores from SD 1 to 85 is shown in Table 2.

**Figure 1:**
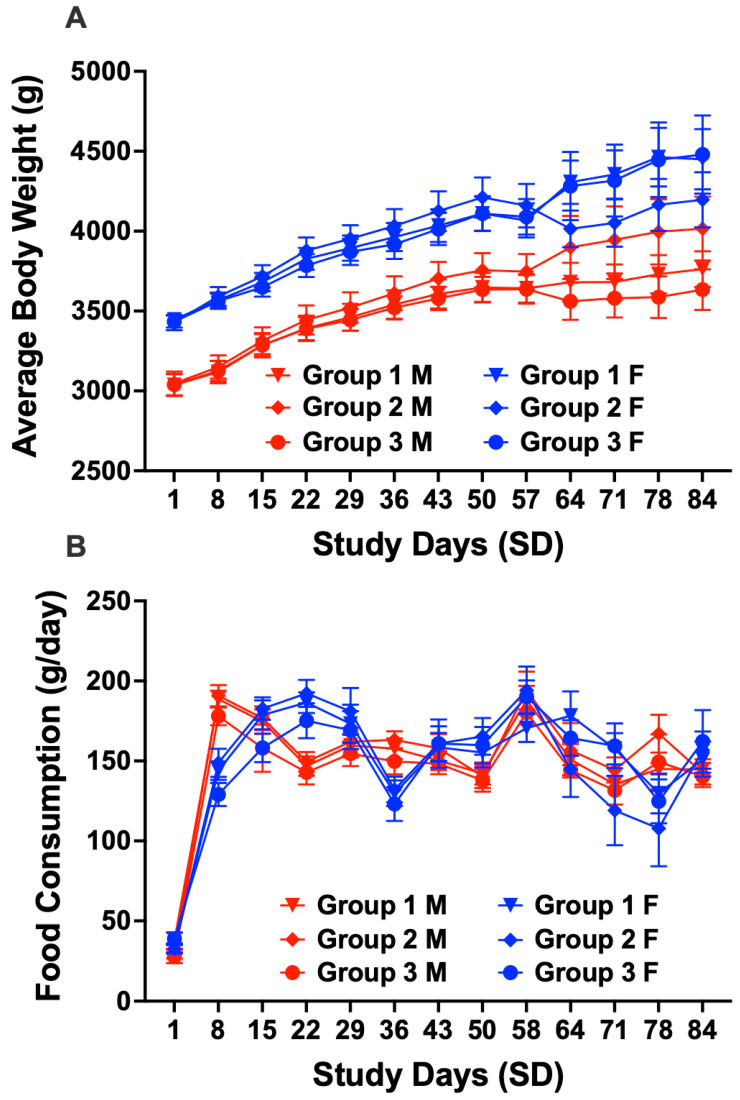
Effect of TT-6-AmHap vaccine on body weight and food consumption. Male (red) and Female (blue) (**A**) body weights were measured prior to first immunization (SD 1); at 24 and 48 ± 1 h after each immunization; and weekly thereafter. (**B**) food consumption was measured daily from arrival. Data are presented as mean ± S.E.M.

Cage side observations of both male and female rabbits showed changes in feces count and consistency. In males, one Group 1, four Group 2, and six Group 3 rabbits had more than one observationof fewer feces from SD 42 to 82, SD 6 to 77, and SD 48 to 82, respectively (Table 2). Similarly in females, two Group 1, two Group 2, and 5 Group 3 rabbits had more than one observation of fewer feces from SD 5 to 5, SD 10 to 47, and SD 5 to 48, respectively (Table 2). Additionally, cage side observations of soft feces were observed in one Group 1 male (on SD 2 and 4), one Group 3 male (on SD 2, 8, and 11), and one Group 3 female (on SD 3 and 24) (Table 2). Because there were no changes in body weights or food consumption (Figure 1), these observations were not considered adverse. Moreover, the vaccines had no effects on body weights or food consumption (Figure 1). The results were comparable across all groups and no statistically significant differences were found.

Treatment with TT-6-AmHap with or without adjuvants had no effect on body temperatures. There were no significant differences noted in body temperatures in male rabbits (Table S1A,B). Vaccinated female rabbits on the other hand exhibited fluctuations in body temperatures, which attained statistical significance (Table S1A,B). Group 2 females exhibited fluctuations in body temperatures on SD 28, 29, 42, 43, and 57, whereas Group 3 females on SD 28 and 57 (Table S1A,B). Additionally, there were some significant increases in body temperatures on dates with no dose administration, specifically SD 28 and 42 (Table S1A,B). Therefore, this is considered incidental and not TT-6-AmHap-related.

Ophthalmology examinations were conducted in all animals at various time points throughout the study. No visible ocular lesions were noted in any of the animals (Table 2). Therefore, there were no TT-6-AmHap-related ocular effects under the conditions of this study.

### 3.2. TT-6-AmHap-Related Effects on Clinical Pathology Parameters

Immunization with TT-6-AmHap with or without adjuvants had no significant effects on the clinical pathology parameters tested. There were no TT-6-AmHap-related effects among the hematology, coagulation, clinical chemistry, and urinalysis endpoints in either sex in any treatment groups compared to the control group (Table S2). With the urinalysis samples, there were some variations between treatment groups among physical (appearance), microscopic, and biochemical urinary components; however, all findings were considered within expected ranges for biological and/or procedure-related variability (Tables S2 and S3).

Furthermore, there was no macroscopic pathology observations related to the administration of the vaccine (Table S4A–D). All macroscopic findings occurred sporadically and were present at a similar incidence in both control and vaccinated animals. Microscopic pathology findings were similar in animals dosed with TT-6-AmHap with or without adjuvants and were limited to the injection sites, commonly observed within the deep muscle layer beneath the subcutaneous tissue (Table S5). There was a slight increase in the sporadic occurrence of minimal to mild inflammation, myofiber necrosis, and/or infiltrates after intramuscular injection of the vaccine in the adjuvanted groups (Group 3) compared to the other groups (Table S5).

### 3.3. TT-6-AmHap-Related Effects on Organ-Specific Toxicity

Organ weight changes at terminal or recovery necropsy for all animals were considered sporadic, independent of dose, small in magnitude, affected only the absolute or relative weights and not both (Table S6A–F). The mean absolute and relative (to body and brain weight) pituitary gland weights were statistically greater in Group 2 males than the controls at the end of the terminal phase, SD 60 (Table S6A). These findings were not noted in female rabbits (Table S6A). There were no vaccine-related organ weight differences at the end of the recovery phase, SD 85. The increased pituitary gland weights present at the terminal necropsy did not persist at the recovery necropsy (Table S6A,D).

### 3.4. Immunogenicity of TT-6-AmHap Vaccine

**Figure 2:**
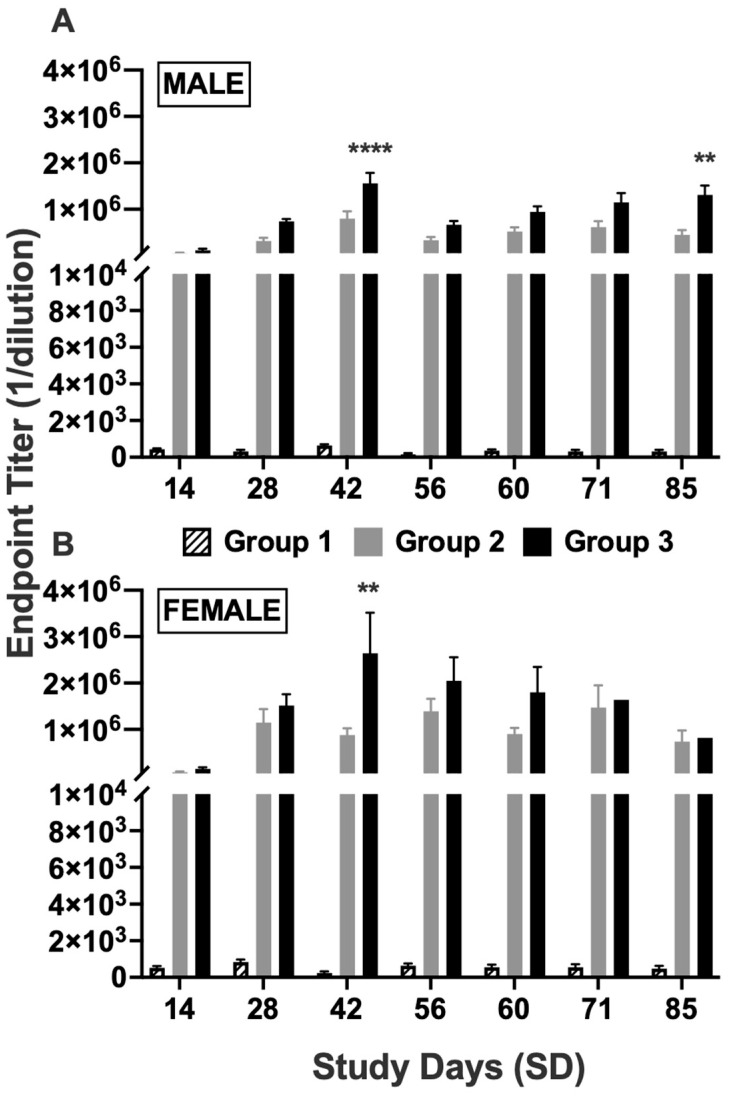
6-AmHap-specific binding antibody response to TT-6-Amhap vaccinations. (**A**) Male and (**B**) Female serum IgG levels after TT-6-AmHap immunization every two weeks over a 57-study-day period. Blood was collected prior to first immunization (SD-4) and on SD 14, 28, 42, 56, 60, 71, and 85. Group 1 (pattern) saline/vehicle control; Group 2 (gray) TT-6-AmHap without adjuvant; Group 3 (black) TT-6-AmHap with adjuvants. Values represent the mean ±S.E.M. of triplicate determinations. Significance of endpoint titers between Groups 2 and 3 was determined by two-way ANOVA with Sidak’s multiple comparisons test. (**A**) ** *p* = 0.008, **** *p* < 0.0001, (**B**) ** *p* = 0.0011; compared to Group 2.

There were no detectable antibody titers to 6-AmHap in Group 1 in both sexes, only baseline levels (Figure 2). Antibody titers to 6-AmHap were elevated in all animals following the administration of TT-6-AmHap with or without adjuvants (Figure 2). Both male and female rabbits from Groups 2 and 3 demonstrated high levels of antibody titers on SD 14 after primary immunization, followed by boosting effects of the vaccine on SD 28 and 42, and remained elevated throughout the duration of the study (Figure 2). Furthermore, female rabbits had an overall better binding antibody titer response compared to the males. Additionally, there was no significant differences in antibody titers in animals immunized with TT-6-AmHap with or without adjuvants on any day post-immunization except for SD 42 and 85 in male rabbits (Figure 2A) and SD 42 in female rabbits (Figure 2B).

## 4. Discussion

In this study, the safety and toxicity of the TT-6-AmHap heroin vaccine with or without adjuvants were evaluated in male and female rabbits. The results of this study indicated that the administration of TT-6-AmHap, with or without adjuvants, did not result in any significant adverse effects on the health and well-being of the test animals. The clinical findings observed following the administration of the TT-6-AmHap vaccine were generally consistent with the safety profiles reported for many other vaccine candidates evaluated in preclinical toxicology studies [27,30,31]. All animals survived until the scheduled time of euthanization. Physical examinations revealed no significant differences between the treated and control groups indicating the lack of overt systemic toxicity or lethality associated with the vaccine, which is a desirable outcome in vaccine safety assessments [32].

While there are limited toxicological profile data on opioid-specific vaccines, the transient local reactions at the injection site (minimal edema and erythema) aligned with the previously published toxicology profile of the oxycodone vaccine, Oxy(Gly)4-sKLH [27]. These localized inflammatory responses were attributed to the body’s initial reaction to the vaccine components, which were quickly resolved. The slight increase in local reactions in the adjuvant group was expected, as adjuvants are specifically included in vaccines to enhance the immune response [12,33,34], often by causing a localized inflammatory milieu [35]. Furthermore, cage-side observations revealed sporadic changes in fecal output across all groups, including the control. The effects of opioid-specific vaccines on gastrointestinal motility have not been investigated in any studies; therefore, this was an important determination since opioids are known to cause a decrease in GI motility [36,37]. The changes in fecal output suggest that these findings were likely related to general environmental or physiological fluctuations observed in laboratory animals rather than being a direct consequence of the TT-6-AmHap vaccine.

Immunization with the TT-6-AmHap vaccine, with or without adjuvants, did not induce any significant effects on the comprehensive panel of clinical pathology parameters evaluated in this study. Hematology, coagulation, clinical chemistry, and urinalysis endpoints remained largely unaffected across all treatment groups and in both sexes. While minor variations were observed in some urinalysis components, they were within the expected range of biological and/or procedure-related variability and were comparable to other toxicology studies in rabbits using other vaccines [30,38], further supporting the tolerability of our vaccine.

Consistent with the clinical pathology observations, the observed changes in organ weights at both the terminal and recovery necropsies were largely considered sporadic and not directly attributable to the vaccine administration. While statistically significant differences in the absolute and relative weights were observed in some organs, several factors suggest these findings are of limited toxicological significance. Firstly, these differences were small in magnitude and not accompanied by corresponding changes in both absolute and relative organ weights, which is often a more reliable indicator of a treatment-related effect [39,40,41]. Secondly, the changes were not consistently observed across both sexes or in the presence or absence of the adjuvants, thus failing to establish a clear relationship with the administration of the TT-6-AmHap vaccine. Finally, the lack of any histopathological correlation in these organs further supports the interpretation that these weight variations were likely within the normal biological variability for rabbits or potentially related to isolated, non-treatment-related factors. These findings are consistent with many toxicology studies evaluating the safety of novel vaccines [27,30,38,42,43,44,45]. Therefore, based on the overall pattern of organ weight data, the TT-6-AmHap vaccine at the tested doses appeared to have a favorable safety profile in rabbits, with no clear evidence of systemic toxicity.

In addition to assessing vaccine safety, the secondary objective of this study was to evaluate the immunogenicity of the TT-6-AmHap vaccine in rabbits and the results clearly demonstrated a robust antibody response following immunization. In contrast to the control group (Group 1), both male and female rabbits in the vaccinated groups (Groups 2 and 3) developed markedly elevated antibody titers to 6-AmHap after the primary immunization on SD 14. This rapid seroconversion indicated that the TT-6-AmHap vaccine was effective in eliciting an initial immune response, which is consistent with previous studies from our laboratory [9,14]. Furthermore, the significant increase in antibody titers observed following the booster immunizations on SD 28 and 42 highlighted the vaccine’s ability to induce immunological memory and enhance the humoral response upon re-exposure to the antigen or drug hapten [46,47]. The sustained elevation of antibody titers throughout the duration of the study suggests a durable and potentially long-lasting antibody half-life, a crucial factor for the efficacy of a vaccine against substance use disorders. We previously demonstrated that 6-AmHap specific antibody titers are durable for 27 weeks in rats [14]. The results of the present study mirror those found in previous preclinical and/or clinical safety studies related to anti-drug vaccines, such as vaccine against cocaine [48,49], nicotine [50,51], and oxycodone [27].

Interestingly, our data revealed a trend towards a more pronounced IgG response in female rabbits compared to their male counterparts, which aligns with what others have found [52,53,54]. While the underlying mechanisms for this sex-specific difference warrant further investigation, it is possible that hormonal influences or other intrinsic biological variations contribute to the enhanced antibody production observed in females [55,56]. Another key observation was the generally comparable antibody titers achieved in animals immunized with TT-6-AmHap with or without adjuvants. While statistically significant differences were noted at specific time points (SD 42 and 85 in males, and SD 42 in females), the overall magnitude of these differences appeared modest and transient. This suggests that the TT-6-AmHap vaccine itself was highly immunogenic in rabbits, and the addition of the adjuvant, at the given human dose, did not consistently provide a substantial boost to the antibody response. Although the study was not designed to look at adjuvant effects, ALFA has shown to have potent adjuvant effects in immunized rabbits with malaria and anthrax antigens [57,58]. Therefore, the sporadic significant differences observed might be attributed to individual animal variability or subtle kinetic effects of the adjuvant at those specific time points.

## 5. Conclusions

In this study, the potential local and systemic toxicity of the TT-6-AmHap heroin vaccine with or without adjuvants ALF43 and Alhydrogel^®^ (ALFA) was evaluated in male and female New Zealand White rabbits. This GLP pharmacology-toxicology study provided compelling evidence for the immunogenicity and tolerability of the TT-6-AmHap vaccine in rabbits. The vaccine elicited a robust and sustained antibody response, with a notable trend towards a stronger response in female rabbits. While the addition of the adjuvant did not consistently enhance the overall antibody titers, the vaccine itself proved to be highly immunogenic. Importantly, the comprehensive toxicology assessment revealed a favorable safety profile, with no significant systemic toxicity and only minimal, transient local reactogenicity. As a result of these promising preclinical findings, an Investigational New Drug (IND) application has been approved by the FDA, and the TT-6-AmHap vaccine is poised to move into Phase 1 clinical trial to evaluate its safety and immunogenicity in humans. The immune assessments will focus on antibody titers, cross-reactivity to opioids and memory B cell populations.

## 6. Patents

Alving, C.R., Matyas, G.R., Mayorov, A.V., Rice, K.C., Iyer, M.R., Cheng, K., Li, F., and Jacobson, A.E. Induction Of Highly Specific Antibodies To A Hapten But Not To A Carrier Peptide By Immunization; US 9,193,739, B2 issued 24 November 2015.

## Figures and Tables

**Table 1:** Experimental design of the TT-6-AmHap toxicology study.

Group	Treatment	Dose Level (μg)	Dose Volume (mL)	Number of Animals			
				Terminal Phase		Recovery Phase	
				Males	Females	Males	Females
1	Vehicle/Control ^a^	0	0.5	5	5	5	5
2	TT-6-AmHap	96	0.5	5	5	5	5
3	TT-6-AmHap +ALF43 +Alhydrogel^®^	96 +171 ^b^ +527 ^c^	0.5	5	5	5	5

^a^ 0.9% Sodium Chloride. ^b^ MPLA (3D-PHAD^®^) (Synthetic MPLA) concentration in liposomes. ^c^ Aluminum concentration.

**Table 2:** Summary of animal observations and measurements.

			Dermal Draize Observations	Physical Examinations ^b^			Cage Side Observations ^c^		Ophthalmology Examinations ^d^
			Injection Site	Clinical Sign					
Group #	Sex	N	Normal ^a^	Abrasions	Discolored Urine	Urine Stain	Few Feces	Soft Feces	Ocular Lesions
1	M	10	8	2	0	0	1	1	0
2	M	10	8	1	2	1	4	0	0
3	M	10	9	2	0	0	6	1	0
1	F	10	10	0	0	0	2	0	0
2	F	10	7	0	0	0	2	0	0
3	F	10	9	0	0	0	5	1	0

^a^ Number of animals with no remarkable observations from SD 1–85. ^b^ Number of animals with abnormal physical examinations from SD 1–85. ^c^ Number of animals with abnormal cage side observations from SD 1–85. ^d^ Number of animals with no visible ocular lesions from SD 1–85.

## Data Availability

Data presented in this study are available from the corresponding author upon request.

## Supplementary Materials

The following supporting information can be downloaded at https://www.mdpi.com/article/10.3390/vaccines13080792/s1. Table S1: Summary of Body Temperature; Table S2: Summary of Clinical Pathology data; Table S3: Summary of Urinalysis Data; Table S4: Summary of Macroscopic Pathology Observations; Table S5: TT-6-AmHap-Related Microscopic Findings—Terminal Phase; Table S6: Summary of Organ Weight Data.

## Abbreviations

The following abbreviations are used in this manuscript:

3D-PHAD®	Monophosphoryl 3-Deacyl Lipid A (Synthetic)Pat No. 9,241,988
6-AmHap	N-((7S,7aR,12bS)-7-acetamido-3-methyl-2,3,4,4a,5,6,7,7a-octahydro-1H-4,12-methanobenzofuro [3,2-e]isoquinolin-9-yl)-3-(tritylthio)propanamide)
ABTS	2,2′-azino-bis(3-ethylbenzothiazoline-6-sulfonic acid
ALF43	Army Liposome Formulation with 43% cholesterol
BSA	Bovine serum albumin
cGMP	Current Good Manufacturing Practice
FDA	Food and Drug Administration
GLP	Good Laboratory Practice
HRP	Horseradish Peroxidase
IgG	Immunoglobulin
MPLA	Monophosphoryl Lipid A
OUD	Opioid use disorder
SD	Study days
S.E.M.	Standard Error of the Mean
TT	tetanus toxoid

## References

[1] Ahmad F.B., Cisewski J.A., Rossen L.M., Sutton P. (2025). Provisional Drug Overdose Death Counts. National Center for Health Statistics. https://www.cdc.gov/nchs/nvss/vsrr/drug-overdose-data.htm.

[2] Volkow N.D., Blanco C. (2021). The Changing Opioid Crisis: Development, Challenges and Opportunities. Mol. Psychiatry.

[3] Leshner A.I., Mancher M., National Academies of Sciences, Engineering, and Medicine (2019). Committee on Medication-Assisted Treatment for Opioid Use Disorder. Medications for Opioid Use Disorder Save Lives.

[4] Tuncturk M., Kushwaha S., Heider R.M., Oesterle T., Weinshilboum R., Ho M.F. (2025). The Development of Opioid Vaccines as a Novel Strategy for the Treatment of Opioid Use Disorder and Overdose Prevention. Int. J. Neuropsychopharmacol..

[5] Janda K.D., Treweek J.B. (2012). Vaccines Targeting Drugs of Abuse: Is the Glass Half-Empty or Half-Full?. Nat. Rev. Immunol..

[6] Clementi M.E., Marini S., Condò S.G., Giardina B. (1991). Antibodies against Small Molecules. Ann. Ist. Super. Sanita.

[7] Landsteiner K., Jacobs J. (1935). Studies on The Sensitization of Animals with Simple Chemical Compounds. J. Exp. Med..

[8] Landsteiner K., van der Scheer J. (1934). On the Serological Specificity of Peptides. II. J. Exp. Med..

[9] Komla E., Torres O.B., Jalah R., Sulima A., Beck Z., Alving C.R., Jacobson A.E., Rice K.C., Matyas G.R. (2021). Effect of Preexisting Immunity to Tetanus Toxoid on the Efficacy of Tetanus Toxoid-Conjugated Heroin Vaccine in Mice. Vaccines.

[10] Jalah R., Torres O.B., Mayorov A.V., Li F., Antoline J.F.G., Jacobson A.E., Rice K.C., Deschamps J.R., Beck Z., Alving C.R. (2015). Efficacy, but Not Antibody Titer or Affinity, of a Heroin Hapten Conjugate Vaccine Correlates with Increasing Hapten Densities on Tetanus Toxoid, but Not on CRM 197 Carriers. Bioconjug. Chem..

[11] Baruffaldi F., Kelcher A.H., Laudenbach M., Gradinati V., Limkar A., Roslawski M., Birnbaum A., Lees A., Hassler C., Runyon S. (2018). Preclinical Efficacy and Characterization of Candidate Vaccines for Treatment of Opioid Use Disorders Using Clinically Viable Carrier Proteins. Mol. Pharm..

[12] Alving C.R., Matyas G.R., Torres O., Jalah R., Beck Z. (2014). Adjuvants for Vaccines to Drugs of Abuse and Addiction. Vaccine.

[13] Beck Z., Matyas G.R., Jalah R., Rao M., Polonis V.R., Alving C.R. (2015). Differential Immune Responses to HIV-1 Envelope Protein Induced by Liposomal Adjuvant Formulations Containing Monophosphoryl Lipid A with or without QS21. Vaccine.

[14] Sulima A., Jalah R., Antoline J.F.G., Torres O.B., Imler G.H., Deschamps J.R., Beck Z., Alving C.R., Jacobson A.E., Rice K.C. (2018). A Stable Heroin Analogue That Can Serve as a Vaccine Hapten to Induce Antibodies That Block the Effects of Heroin and Its Metabolites in Rodents and That Cross-React Immunologically with Related Drugs of Abuse. J. Med. Chem..

[15] Booy R., Heath P.T., Slack M.P., Begg N., Richard Moxon E. (1997). Vaccine Failures after Primary Immunisation with Haemophilus Influenzae Type-b Conjugate Vaccine without Booster. Lancet.

[16] Lieberman J.M., Greenberg D.P., Wong V.K., Partridge S., Chang S.-J., Chiu C.-Y., Ward J.I. (1995). Effect of Neonatal Immunization with Diphtheria and Tetanus Toxoids on Antibody Responses to Haemophilus Influenzae Type b Conjugate Vaccines. J. Pediatr..

[17] Barington T., Kristensen K., Henrichsen J., Heilmann C. (1991). Influence of Prevaccination Immunity on the Human B-Lymphocyte Response to a Haemophilus Influenzae Type b Conjugate Vaccine. Infect. Immun..

[18] Barington T., Skettrup M., Juul L., Heilmann C. (1993). Non-Epitope-Specific Suppression of the Antibody Response to Haemophilus Influenzae Type b Conjugate Vaccines by Preimmunization with Vaccine Components. Infect. Immun..

[19] Barrientos R.C., Whalen C., Torres O.B., Sulima A., Bow E.W., Komla E., Beck Z., Jacobson A.E., Rice K.C., Matyas G.R. (2021). Bivalent Conjugate Vaccine Induces Dual Immunogenic Response That Attenuates Heroin and Fentanyl Effects in Mice. Bioconjug. Chem..

[20] Torres O.B., Jalah R., Rice K.C., Li F., Antoline J.F.G., Iyer M.R., Jacobson A.E., Boutaghou M.N., Alving C.R., Matyas G.R. (2014). Characterization and Optimization of Heroin Hapten-BSA Conjugates: Method Development for the Synthesis of Reproducible Hapten-Based Vaccines. Anal. Bioanal. Chem..

[21] Ban B., Barrientos R.C., Oertel T., Komla E., Whalen C., Sopko M., You Y., Banerjee P., Sulima A., Jacobson A.E. (2021). Novel Chimeric Monoclonal Antibodies That Block Fentanyl Effects and Alter Fentanyl Biodistribution in Mice. MAbs.

[22] Pravetoni M., Le Naour M., Harmon T.M., Tucker A.M., Portoghese P.S., Pentel P.R. (2012). An Oxycodone Conjugate Vaccine Elicits Drug-Specific Antibodies That Reduce Oxycodone Distribution to Brain and Hot-Plate Analgesia. J. Pharmacol. Exp. Ther..

[23] Raleigh M.D., Baruffaldi F., Peterson S.J., Le Naour M., Harmon T.M., Vigliaturo J.R., Pentel P.R., Pravetoni M. (2019). A Fentanyl Vaccine Alters Fentanyl Distribution and Protects against Fentanyl-Induced Effects in Mice and Rats. J. Pharmacol. Exp. Ther..

[24] Tenney R.D., Blake S., Bremer P.T., Zhou B., Hwang C.S., Poklis J.L., Janda K.D., Banks M.L. (2019). Vaccine Blunts Fentanyl Potency in Male Rhesus Monkeys. Neuropharmacology.

[25] Townsend E.A., Blake S., Faunce K.E., Hwang C.S., Natori Y., Zhou B., Bremer P.T., Janda K.D., Banks M.L. (2019). Conjugate Vaccine Produces Long-Lasting Attenuation of Fentanyl vs. Food Choice and Blocks Expression of Opioid Withdrawal-Induced Increases in Fentanyl Choice in Rats. Neuropsychopharmacology.

[26] Bremer P.T., Schlosburg J.E., Banks M.L., Steele F.F., Zhou B., Poklis J.L., Janda K.D. (2017). Development of a Clinically Viable Heroin Vaccine. J. Am. Chem. Soc..

[27] Hamid F.A., Marker C.L., Raleigh M.D., Khaimraj A., Winston S., Pentel P.R., Pravetoni M. (2022). Pre-Clinical Safety and Toxicology Profile of a Candidate Vaccine to Treat Oxycodone Use Disorder. Vaccine.

[28] Matyas G.R., Muderhwa J.M., Alving C.R. (2003). Oil-in-Water Liposomal Emulsions for Vaccine Delivery. Methods Enzymol..

[29] Beck Z., Torres O.B., Matyas G.R., Lanar D.E., Alving C.R. (2018). Immune Response to Antigen Adsorbed to Aluminum Hydroxide Particles: Effects of Co-Adsorption of ALF or ALFQ Adjuvant to the Aluminum-Antigen Complex. J. Control. Release.

[30] Park S.J., Jang M.S., Lim K.H., Seo J.W., Im W.J., Han K.H., Kim S.E., Jang E., Park D., Kim Y.B. (2023). Preclinical Evaluation of General Toxicity and Safety Pharmacology of a Receptor-Binding Domain-Based COVID-19 Subunit Vaccine in Various Animal Models. Arch. Toxicol..

[31] Yang H., Pan W., Chen G., Huang E., Lu Q., Chen Y., Chen Y., Yang Z., Wen L., Zhang S. (2022). Preclinical Toxicity and Immunogenicity of a COVID-19 Vaccine (ZF2001) in Cynomolgus Monkeys. Vaccines.

[32] Suzumura Y. (2024). Importance of Examining Incidentality in Vaccine Safety Assessment. Vaccines.

[33] Alving C.R., Peachman K.K., Rao M., Reed S.G. (2012). Adjuvants for Human Vaccines. Curr. Opin. Immunol..

[34] Reed S.G., Orr M.T., Fox C.B. (2013). Key Roles of Adjuvants in Modern Vaccines. Nat. Med..

[35] Coffman R.L., Sher A., Seder R.A. (2010). Vaccine Adjuvants: Putting Innate Immunity to Work. Immunity.

[36] Akbarali H.I., Dewey W.L. (2019). Gastrointestinal Motility, Dysbiosis and Opioid-Induced Tolerance: Is There a Link?. Nat. Rev. Gastroenterol. Hepatol..

[37] Chan L.-N. (2008). Opioid Analgesics and the Gastrointestinal Tract. Pract. Gastroenterol..

[38] Ramot Y., Kronfeld N., Ophir Y., Ezov N., Friedman S., Saloheimo M., Vitikainen M., Ben-Artzi H., Avigdor A., Tchelet R. (2022). Toxicity and Local Tolerance of a Novel Spike Protein RBD Vaccine Against SARS-CoV-2, Produced Using the C1 *Thermothelomyces heterothallica* Protein Expression Platform. Toxicol. Pathol..

[39] Michael B., Yano B., Sellers R.S., Perry R., Morton D., Roome N., Johnson J.K., Schafer K., Pitsch S. (2007). Evaluation of Organ Weights for Rodent and Non-Rodent Toxicity Studies: A Review of Regulatory Guidelines and a Survey of Current Practices. Toxicol. Pathol..

[40] Mezencev R., Feshuk M., Kolaczkowski L., Peterson G.C., Zhao Q.J., Watford S., Weaver J.A. (2024). The Association between Histopathologic Effects and Liver Weight Changes Induced in Mice and Rats by Chemical Exposures: An Analysis of the Data from Toxicity Reference Database (ToxRefDB). Toxicol. Sci..

[41] Sellers R.S., Morton D., Michael B., Roome N., Johnson J.K., Yano B.L., Perry R., Schafer K. (2007). Society of Toxicologic Pathology Position Paper: Organ Weight Recommendations for Toxicology Studies. Toxicol. Pathol..

[42] Dai X., Zhao W., Tong X., Liu W., Zeng X., Duan X., Wu H., Wang L., Huang Z., Tang X. (2022). Non-Clinical Immunogenicity, Biodistribution and Toxicology Evaluation of a Chimpanzee Adenovirus-Based COVID-19 Vaccine in Rat and Rhesus Macaque. Arch. Toxicol..

[43] Rohde C.M., Lindemann C., Giovanelli M., Sellers R.S., Diekmann J., Choudhary S., Ramaiah L., Vogel A.B., Chervona Y., Muik A. (2023). Toxicological Assessments of a Pandemic COVID-19 Vaccine—Demonstrating the Suitability of a Platform Approach for MRNA Vaccines. Vaccines.

[44] Nurpeisova A., Khairullin B., Abitaev R., Shorayeva K., Jekebekov K., Kalimolda E., Kerimbayev A., Akylbayeva K., Abay Z., Myrzakhmetova B. (2022). Safety and Immunogenicity of the First Kazakh Inactivated Vaccine for COVID-19. Hum. Vaccines Immunother..

[45] Tusé D., Malm M., Tamminen K., Diessner A., Thieme F., Jarczowski F., Blazevic V., Klimyuk V. (2022). Safety and Immunogenicity Studies in Animal Models Support Clinical Development of a Bivalent Norovirus-like Particle Vaccine Produced in Plants. Vaccine.

[46] Plotkin S.A. (2003). Vaccines, Vaccination, and Vaccinology. J. Infect. Dis..

[47] Plotkin S.A. (2008). Correlates of Vaccine-Induced Immunity. Clin. Infect. Dis..

[48] Sabato B., Augusto P.S.d.A., Pereira R.L.G., Esteves F.C.B., Caligiorne S.M., Assis B.R.D., Marcelino S.A.C., Santo L.P.D.E., dos Reis K.D., Neto L.D.S. (2023). Safety and Immunogenicity of the Anti-Cocaine Vaccine UFMG-VAC-V4N2 in a Non-Human Primate Model. Vaccine.

[49] Kosten T.R., Rosen M., Bond J., Settles M., Roberts J.S.C., Shields J., Jack L., Fox B. (2002). Human Therapeutic Cocaine Vaccine: Safety and Immunogenicity. Vaccine.

[50] Maurer P., Jennings G.T., Willers J., Rohner F., Lindman Y., Roubicek K., Renner W.A., Müller P., Bachmann M.F. (2005). A Therapeutic Vaccine for Nicotine Dependence: Preclinical Efficacy, and Phase I Safety and Immunogenicity. Eur. J. Immunol..

[51] Hoogsteder P.H., Kotz D., Van Spiegel P.I., Viechtbauer W., Brauer R., Kessler P.D., Kalnik M.W., Fahim R.E., Van Schayck O.C. (2012). The Efficacy and Safety of a Nicotine Conjugate Vaccine (NicVAX^®^) or Placebo Co-Administered with Varenicline (Champix^®^) for Smoking Cessation: Study Protocol of a Phase IIb, Double Blind, Randomized, Placebo Controlled Trial. BMC Public Health.

[52] Fischinger S., Boudreau C.M., Butler A.L., Streeck H., Alter G. (2019). Sex Differences in Vaccine-Induced Humoral Immunity. Semin. Immunopathol..

[53] St Clair L.A., Chaulagain S., Klein S.L., Benn C.S., Flanagan K.L. (2023). Sex-Differential and Non-Specific Effects of Vaccines Over the Life Course. Current Topics in Microbiology and Immunology.

[54] Klein S.L., Marriott I., Fish E.N. (2014). Sex-Based Differences in Immune Function and Responses to Vaccination. Trans. R. Soc. Trop. Med. Hyg..

[55] Klein S.L., Flanagan K.L. (2016). Sex Differences in Immune Responses. Nat. Rev. Immunol..

[56] Ortona E., Pierdominici M., Rider V. (2019). Editorial: Sex Hormones and Gender Differences in Immune Responses. Front. Immunol..

[57] Richards R.L., Hayre M.D., Hockmeyer W.T., Alving C.R. (1988). Liposomes, Lipid A, and Aluminium Hydroxide Enhance the Immune Response to a Synthetic Malaria Sporozoite Antigen. Infect. Immun..

[58] Richards R.L., Alving C.R., Wassef N.M. (1996). Liposomal Subunit Vaccines: Effects of Lipid A and Aluminum Hydroxide on Immunogenicity. J. Pharm. Sci..

