# Supplementary material for: Safety and Toxicology Profile of TT-6-AmHap Heroin Conjugate Vaccine
Source: Vaccines (Basel). 2025 Jul 26;13(8):792. doi: 10.3390/vaccines13080792 (PMC12390132; doi:10.3390/vaccines13080792)
Supplement: Supplementary file 1 [file vaccines-13-00792-s001.zip › vaccines-3755108-supplementary.pdf]

Safety and toxicology profile of TT-6-AmHap heroin conjugate vaccine

Essie Komla [1,2], Erwin G. Abucayon [1,2,a], C. Steven Godin [3], Agnieszka Sulima [4], Arthur E. Jacobson [4], Kenner C. Rice [4], and Gary R. Matyas [1,*]

Supplemental Tables

Table S1A: Summary of Body Temperature (°C)

Day Numbers Relative to Start Date

Group	Sex		-1	1<br>3.0HR	1<br>6.0HR	2	14	15<br>3.0HR	15<br>6.0HR	16	28	29<br>3.0HR
1	m	Mean	38.96	39.05	39.10	38.54	38.49	38.73	39.03	38.89	38.53	38.87
		S.D.	0.23	0.37	0.48	0.38	0.39	0.40	0.34	0.38	0.37	0.52
		N	10	10	10	10	10	10	10	10	10	10
2	m	Mean	38.59	38.79	38.82	38.36	38.49	38.78	38.89	38.69	38.45	38.65
		S.D.	0.34	0.12	0.32	0.33	0.27	0.25	0.28	0.26	0.41	0.18
		N	10	10	10	10	10	10	10	10	10	10
3	m	Mean	38.70	38.96	39.16	38.70	38.75	39.01	39.11	38.75	38.91	38.90
		S.D.	0.28	0.42	0.39	0.26	0.30	0.37	0.35	0.21	0.55	0.35
		N	10	10	10	10	10	10	10	10	10	10
1	f	Mean	38.79	38.79	38.88	38.72	38.57	38.87	39.11	38.75	38.57	38.81
		S.D.	0.30	0.36	0.24	0.32	0.34	0.24	0.18	0.22	0.36	0.34
		N	10	10	10	10	10	10	10	10	10	10
2	f	Mean	38.88	38.99	39.07	38.96	38.82	39.09	39.28	38.87	38.90*	39.18*
		S.D.	0.22	0.30	0.18	0.18	0.21	0.29	0.23	0.18	0.28	0.27
		N	10	10	10	10	10	10	10	10	10	10
3	f	Mean	38.82	38.96	39.01	38.83	38.78	39.08	39.30	38.86	38.89*	39.04
		S.D.	0.29	0.25	0.23	0.22	0.25	0.37	0.28	0.28	0.26	0.21
		N	10	10	10	10	10	10	10	10	10	10

* - Significantly different from the control value, $p \leq 0.05$

Nominal Dose: Group 1 – 0 µg Group 2 – 96 µg Group 3 - 96+171+527 µg

Table S1B: Summary of Body Temperature (°C)

Group	Sex		Day 29 6.0HR	30	42	43 3.0HR	43 6.0HR	44	56	57 3.0HR	57 6.0HR	58
1	m	Mean	39.10	38.72	38.59	38.94	39.11	38.70	38.35	38.85	39.27	38.78
		S.D.	0.44	0.47	0.48	0.57	0.45	0.48	0.51	0.38	0.45	0.36
		N	10	10	10	10	10	10	10	10	10	10
2	m	Mean	38.84	38.48	38.20	38.64	38.88	38.41	38.17	38.64	38.84	38.51
		S.D.	0.19	0.23	0.33	0.32	0.28	0.30	0.28	0.33	0.21	0.41
		N	10	10	10	10	10	10	10	10	10	10
3	m	Mean	39.01	38.77	38.60	38.89	39.05	38.64	38.42	38.87	39.14	38.80
		S.D.	0.30	0.40	0.50	0.36	0.44	0.54	0.34	0.43	0.53	0.53
		N	10	10	10	10	10	10	10	10	10	10
1	f	Mean	39.03	38.77	38.64	38.99	38.90	38.69	38.59	38.81	38.67	38.97
		S.D.	0.22	0.26	0.34	0.26	0.33	0.26	0.32	0.38	0.26	0.18
		N	10	10	10	10	10	10	10	10	10	10
2	f	Mean	39.30*	38.87	39.10*	39.21	39.22*	38.89	38.60	39.12	39.25*	39.02
		S.D.	0.18	0.18	0.24	0.20	0.22	0.20	0.15	0.23	0.30	0.16
		N	10	10	10	10	10	10	10	10	10	10
3	f	Mean	39.23	38.89	38.88	39.23	39.15	38.76	38.63	39.25*	39.10*	39.02
		S.D.	0.21	0.19	0.31	0.33	0.25	0.28	0.37	0.29	0.40	0.29
		N	10	10	10	10	10	10	10	10	10	10

Day Numbers Relative to Start Date

* - Significantly different from the control value, $p \leq 0.05$

Nominal Dose: Group 1 – 0 µg Group 2 – 96 µg Group 3 - 96+171+527 µg

Table S2: Summary of Clinical Pathology data

				Clinical Pathology							
				Hematology			Coagulation	Clinical Chemistry			Urinalysis
Group #	Sex	Day Number		RBC (M/µL)	WBC (K/µL)	PLT (K/µL)	FIB (mg/dL)	GLU (mg/dL)	BUN (mg/dL)	CRE2 (mg/dL)	URO (U/dL)
1	M	60	Mean	6.15	7.31	298.00	327.50	134.60	12.70	0.84	0.76
			SD	0.36	1.77	37.40	296.40	7.20	1.90	0.10	0.39
			N	10	10	10	10	10	10	10	10
		85	Mean	6.41	6.00	304.00	296.40	128.60	15.80	0.84	0.36
			SD	0.26	0.81	54.80	27.10	5.70	1.90	0.09	0.36
			N	5	5	5	5	5	5	5	5
2	M	60	Mean	6.18	7.16	320.10	333.20	134.10	13.30	0.88	0.38
			SD	0.23	1.37	86.10	49.20	5.20	1.50	0.09	0.35
			N	10	10	10	10	10	10	10	9
		85	Mean	6.48	5.71	307.30	287.00	129.20	17.00	0.86	0.68
			SD	0.17	1.13	77.70	53.50	10.80	1.00	0.09	0.44
			N	5	5	5	5	5	5	5	5
3	M	60	Mean	6.05	7.01	370.20	382.10	134.90	14.10	0.91	0.56
			SD	0.34	1.18	75.40	56.70	6.70	2.70	0.14	0.42
			N	10	10	10	10	10	10	10	9
		85	Mean	6.07	5.89	401.80	295.00	130.40	18.60	0.96	0.68
			SD	0.28	0.89	65.60	73.80	15.20	5.60	0.26	0.44
			N	5	5	5	5	5	5	5	5
1	F	60	Mean	6.09	6.60	303.90	239.70	128.80	17.60	1.27	0.76
			SD	0.46	1.19	113.10	20.10	7.80	1.80	0.18	0.39
			N	10	10	10	10	10	10	10	10
		85	Mean	6.13	5.23	394.40	214.40	119.00	24.20	1.16	0.52
			SD	0.47	0.91	118.00	12.80	4.60	2.70	0.05	0.44
			N	5	5	5	5	5	5	5	5
2	F	60	Mean	5.99	7.70	326.90	299.90	125.40	17.40	1.20	0.52
			SD	0.25	1.87	83.10	56.50	5.20	1.30	0.16	0.41
			N	10	10	10	10	10	10	10	10
		85	Mean	6.1	5.6	386.6	218.8	116.2	23.4	1.3	0.7
			SD	0.3	0.5	113.7	15.6	4.6	2.1	0.2	0.4
			N	5	5	5	5	5	5	5	5
3	F	60	Mean	5.89	6.82	277.00	264.20	124.60	17.20	1.16	0.56
			SD	0.44	1.65	44.20	27.90	4.20	1.50	0.08	0.42
			N	10	10	10	10	10	10	10	9
		85	Mean	5.88	5.16	337.80	203.40	120.00	23.20	1.16	0.68
			SD	0.30	0.76	53.10	17.50	5.70	3.60	0.05	0.44
			N	5	5	5	5	5	5	5	5

**Abbreviation**	**Description**
RBC	Red Blood Cells
WBC	White Blood Cells
PLT	Platelets
FIB	Fibrinogen
GLU	Glucose
BUN	Blood Urea Nitrogen
CRE2	Creatinine
URO	Urobilinogen

Table S3: Summary of Urinalysis Data

Day Numbers Relative to Start Date

Group	Sex	Day Number		SG	UpH	URO U/dL	Group	Sex	Day Number		SG	UpH	URO U/dL
1	m	60	Mean	1.0206	8.05	0.76	1	f	60	Mean	1.0223	7.60	0.76
			S.D.	0.0163	0.37	0.39				S.D.	0.0140	0.84	0.39
			N	10	10	10				N	9	10	10
		85	Mean	1.0082	8.20	0.36			85	Mean	1.0253	8.10	0.52
			S.D.	0.0066	0.45	0.36				S.D.	0.0151	0.55	0.44
			N	5	5	5				N	3	5	5
2	m	60	Mean	1.0144	7.56*	0.38	2	f	60	Mean	1.0094	7.75	0.52
			S.D.	0.0116	0.39	0.35				S.D.	0.0143	0.59	0.41
			N	8	9	9				N	10	10	10
		85	Mean	1.0216	8.00	0.68			85	Mean	1.0303	8.20	0.68
			S.D.	0.0129	0.71	0.44				S.D.	0.0139	0.27	0.44
			N	5	5	5				N	3	5	5
3	m	60	Mean	1.0227	7.94	0.56	3	f	60	Mean	1.0092	7.56	0.56
			S.D.	0.0169	0.68	0.42				S.D.	0.0135	0.68	0.42
			N	9	9	9				N	9	9	9
		85	Mean	1.0198	7.60	0.68			85	Mean	1.0303	7.30	0.68
			S.D.	0.0124	1.02	0.44				S.D.	0.0127	1.30	0.44
			N	5	5	5				N	4	5	5

* - Significantly different from the control value, $p \leq 0.05$

Nominal Dose: Group 1 – 0 µg Group 2 – 96 µg Group 3 - 96+171+527 µg
SG - Specific Gravity, UpH - Urine pH, URO – Urobilinogen

<u>Table S4A: Summary of Macroscopic Pathology Observations</u>

Day Numbers Relative to Start Date

Terminal Necropsy: SD 60

	MALES			FEMALES		
Group:	1	2	3	1	2	3
Number of Animals:	(5)	(5)	(5)	(5)	(5)	(5)
eye;						
Submitted	(5)	(5)	(5)	(5)	(5)	(5)
No Visible Lesions	5	5	5	5	5	5
gallbladder;						
Submitted	(5)	(5)	(5)	(5)	(5)	(5)
No Visible Lesions	5	5	5	5	5	5
gland, adrenal;						
Submitted	(5)	(5)	(5)	(5)	(5)	(5)
No Visible Lesions	5	5	5	5	5	5
gland, mammary;						
Submitted	(5)	(5)	(5)	(5)	(5)	(5)
No Visible Lesions	5	5	5	5	5	5
gland, parathyroid;						
Submitted	(5)	(5)	(5)	(5)	(5)	(5)
No Visible Lesions	5	5	5	5	5	5
gland, pituitary;						
Submitted	(5)	(5)	(5)	(5)	(5)	(5)
No Visible Lesions	5	5	5	5	5	5
gland, prostate;						
Submitted	(5)	(5)	(5)	(-)	(-)	(-)
No Visible Lesions	5	5	5	-	-	-
gland, salivary, submandibular;						
Submitted	(5)	(5)	(5)	(5)	(5)	(5)
No Visible Lesions	5	5	5	5	5	5

Nominal Dose: Group 1 – 0 µg Group 2 – 96 µg Group 3 - 96+171+527 µg

Table S4B: Summary of Macroscopic Pathology Observations

Day Numbers Relative to Start Date

Terminal Necropsy: SD 60

	MALES			FEMALES		
Group:	1	2	3	1	2	3
Number of Animals:	(5)	(5)	(5)	(5)	(5)	(5)
testis;						
Submitted	(5)	(5)	(5)	(-)	(-)	(-)
No Visible Lesions	5	5	5	-	-	-
thymus;						
Submitted	(5)	(5)	(5)	(5)	(5)	(5)
No Visible Lesions	5	5	5	5	5	5
trachea;						
Submitted	(5)	(5)	(5)	(5)	(5)	(5)
No Visible Lesions	5	5	5	5	5	5
urinary bladder;						
Submitted	(5)	(5)	(5)	(5)	(5)	(5)
No Visible Lesions	5	5	5	5	5	5
uterus;						
Submitted	(-)	(-)	(-)	(5)	(5)	(5)
No Visible Lesions	-	-	-	5	5	5
vagina;						
Submitted	(-)	(-)	(-)	(5)	(5)	(5)
No Visible Lesions	-	-	-	5	5	5

Nominal Dose: Group 1 – 0 µg Group 2 – 96 µg Group 3 - 96+171+527 µg

Table S4C: Summary of Macroscopic Pathology Observations

Day Numbers Relative to Start Date

Recovery Necropsy: SD 85

	MALES 1	MALES 2	MALES 3	FEMALES 1	FEMALES 2	FEMALES 3
Group:	1	2	3	1	2	3
Number of Animals:	(5)	(5)	(5)	(5)	(5)	(5)
eye;						
Submitted	(5)	(5)	(5)	(5)	(5)	(5)
No Visible Lesions	5	5	5	5	5	5
gallbladder;						
Submitted	(5)	(5)	(5)	(5)	(5)	(5)
No Visible Lesions	5	5	5	5	5	5
gland, adrenal;						
Submitted	(5)	(5)	(5)	(5)	(5)	(5)
No Visible Lesions	5	5	5	5	5	5
gland, mammary;						
Submitted	(5)	(5)	(5)	(5)	(5)	(5)
No Visible Lesions	5	5	5	5	5	5
gland, parathyroid;						
Submitted	(5)	(5)	(5)	(5)	(5)	(5)
No Visible Lesions	5	5	5	5	5	5
gland, pituitary;						
Submitted	(5)	(5)	(5)	(5)	(5)	(5)
No Visible Lesions	5	5	5	5	5	5
gland, prostate;						
Submitted	(5)	(5)	(5)	(-)	(-)	(-)
No Visible Lesions	5	5	5	-	-	-
gland, salivary, submandibular;						
Submitted	(5)	(5)	(5)	(5)	(5)	(5)
No Visible Lesions	5	5	5	5	5	5

Nominal Dose: Group 1 – 0 µg Group 2 – 96 µg Group 3 - 96+171+527 µg

Table S4D: Summary of Macroscopic Pathology Observations

Day Numbers Relative to Start Date

Recovery Necropsy: SD 85

	MALES			FEMALES		
Group:	1	2	3	1	2	3
Number of Animals:	(5)	(5)	(5)	(5)	(5)	(5)
stomach;						
Submitted	(5)	(5)	(5)	(5)	(5)	(5)
No Visible Lesions	5	5	5	5	5	5
testis;						
Submitted	(5)	(5)	(5)	(-)	(-)	(-)
No Visible Lesions	5	5	5	-	-	-
thymus;						
Submitted	(5)	(5)	(5)	(5)	(5)	(5)
No Visible Lesions	5	5	5	5	5	5
trachea;						
Submitted	(5)	(5)	(5)	(5)	(5)	(5)
No Visible Lesions	5	5	5	5	5	5
urinary bladder;						
Submitted	(5)	(5)	(5)	(5)	(5)	(5)
No Visible Lesions	5	5	5	5	5	5
uterus;						
Submitted	(-)	(-)	(-)	(5)	(5)	(5)
No Visible Lesions	-	-	-	5	5	5
vagina;						
Submitted	(-)	(-)	(-)	(5)	(5)	(5)
No Visible Lesions	-	-	-	5	5	5

Nominal Dose: Group 1 – 0 µg Group 2 – 96 µg Group 3 - 96+171+527 µg

Table S5: TT-6-AmHap-Related Microscopic Findings – Terminal Phase

Sex	Males			Females		
Group	1	2	3	1	2	3
Dose Level (µg)	0	96	96+171+ 527	0	96	96+171+ 527
Number Examined	5	5	5	5	5	5
Injection Site 2 (Day 15)						
INFILTRATE, MIXED CELL						
Minimal	0	1	0	0	1	1
Injection Site 3 (Day 29)						
INFLAMMATION, MIXED CELL						
Minimal	0	0	1	0	0	0
Mild	0	0	1	0	0	0
NECROSIS; Myofiber						
Mild	0	0	1	0	0	0
INFILTRATE, MIXED CELL						
Minimal	0	0	0	0	1	1
Injection Site 4 (Day 43)						
INFILTRATE, MIXED CELL						
Minimal	0	1	0	1	0	2
Mild	0	0	0	0	0	1
CELLULARITY, INCREASED; Macrophage						
Mild	0	0	1	0	0	0
Injection Site 5 (Day 57)						
INFILTRATE, MIXED CELL						
Minimal	0	2	1	1	1	2
INFLAMMATION, MIXED CELL						
Minimal	0	0	2	0	0	1
Mild	0	0	0	0	0	1
HEMORRHAGE						
Minimal	0	0	1	1	0	0
NECROSIS; Myofiber						
Minimal	0	0	1	0	0	1
FIBROSIS						
Minimal	0	1	0	1	0	0

Table S6A: Summary of Organ Weight Data

Day 60 Relative to Start Date

Group	Sex		Kidneys Abs	Kidneys /BW	Kidneys /BR	Liver/Gallbladder Abs	Liver/Gallbladder /BW	Liver/Gallbladder /BR	Pituitary Abs	Pituitary /BW	Pituitary /BR
1	m	Mean	18.2514	0.51336	1.99388	92.7966	2.61754	10.13586	0.02538	0.00070	0.00278
		S.D.	3.1685	0.08679	0.38902	17.1240	0.50909	2.08569	0.00266	0.00012	0.00029
		N	5	5	5	5	5	5	5	5	5
2	m	Mean	18.2152	0.51344	1.98988	93.8438	2.64626	10.23036	0.03832*	0.00108*	0.00416*
		S.D.	2.5928	0.04006	0.32583	14.4402	0.27419	1.62092	0.01247	0.00036	0.00123
		N	5	5	5	5	5	5	5	5	5
3	m	Mean	18.4670	0.50744	1.93810	91.6696	2.52702	9.64408	0.03174	0.00090	0.00332
		S.D.	1.6346	0.03204	0.21385	7.0254	0.25581	1.20378	0.01001	0.00034	0.00099
		N	5	5	5	5	5	5	5	5	5
1	f	Mean	14.7830	0.38664	1.64540	71.8678	1.88550	7.95686	0.02646	0.00072	0.00288
		S.D.	2.7530	0.04894	0.36753	9.9121	0.17113	1.22217	0.00919	0.00026	0.00085
		N	5	5	5	5	5	5	5	5	5
2	f	Mean	17.9462*	0.42228	1.95174	86.3058	2.02728	9.36554	0.04070	0.00094	0.00428
		S.D.	0.9713	0.02380	0.25997	9.6773	0.19131	1.35354	0.01284	0.00031	0.00113
		N	5	5	5	5	5	5	5	5	5
3	f	Mean	16.3382	0.41920	1.70486	72.8506	1.86362	7.56544	0.04002	0.00104	0.00416
		S.D.	1.2381	0.03705	0.35196	9.8586	0.22361	1.52022	0.01831	0.00054	0.00197
		N	5	5	5	5	5	5	5	5	5

* - Significantly different from the control value, $p \leq 0.05$

Nominal Dose: Group 1 – 0 µg Group 2 – 96 µg Group 3 - 96+171+527 µg

Absolute organ weights in grams (Abs), organ-to-body weight ratios (/BW), and organ-to-brain weight ratios (/BR)

Table S6B: Summary of Organ Weight Data

Day 60 Relative to Start Date

Group	Sex		Spleen Abs	Spleen /BW	Spleen /BR	Thymus Abs	Thymus /BW	Thymus /BR	Thyroid/Parathyroid Abs	Thyroid/Parathyroid /BW	Thyroid/Parathyroid /BR
1	m	Mean	1.1892	0.03354	0.12974	4.8614	0.13604	0.53158	0.26968	0.00756	0.02946
		S.D.	0.1331	0.00424	0.01702	1.7933	0.04625	0.20541	0.04921	0.00114	0.00582
		N	5	5	5	5	5	5	5	5	5
2	m	Mean	1.1600	0.03314	0.12624	5.6164	0.15870	0.61120	0.23674	0.00680	0.02594
		S.D.	0.1433	0.00580	0.01487	1.5550	0.04251	0.16513	0.07179	0.00236	0.00846
		N	5	5	5	5	5	5	5	5	5
3	m	Mean	1.1884	0.03272	0.12558	4.9032	0.13438	0.52346	0.24006	0.00654	0.02520
		S.D.	0.2681	0.00711	0.03548	1.3497	0.03471	0.17968	0.06147	0.00140	0.00641
		N	5	5	5	5	5	5	5	5	5
1	f	Mean	1.5642	0.04150	0.17244	4.1570	0.10914	0.46882	0.24568	0.00648	0.02712
		S.D.	0.2149	0.00781	0.01838	1.4255	0.03640	0.19238	0.07381	0.00213	0.00810
		N	5	5	5	5	5	5	5	5	5
2	f	Mean	2.2566	0.05144	0.23602	4.9014	0.11494	0.51316	0.31782	0.00736	0.03318
		S.D.	1.2435	0.02206	0.10063	1.8690	0.04183	0.16395	0.13631	0.00260	0.01080
		N	5	5	5	5	5	5	5	5	5
3	f	Mean	1.4616	0.03734	0.14706	4.4002	0.11318	0.46212	0.36878	0.00948	0.03840
		S.D.	0.4453	0.01066	0.02743	0.7816	0.02230	0.12858	0.02277	0.00083	0.00713
		N	5	5	5	5	5	5	5	5	5

Nominal Dose: Group 1 – 0 µg Group 2 – 96 µg Group 3 - 96+171+527 µg
Absolute organ weights in grams (Abs), organ-to-body weight ratios (/BW), and organ-to-brain weight ratios (/BR)

Table S6C: Summary of Organ Weight Data

Day 60 Relative to Start Date

Group	Sex		Epididymides Abs	Epididymides /BW	Epididymides /BR	Testes Abs	Testes /BW	Testes /BR
1	m	Mean	3.0906	0.08742	0.33632	5.5162	0.15572	0.60094
		S.D.	0.1697	0.01054	0.01118	0.4446	0.01700	0.05246
		N	5	5	5	5	5	5
2	m	Mean	3.0046	0.08556	0.32526	5.5046	0.15486	0.60354
		S.D.	0.5324	0.01804	0.04209	1.1136	0.02244	0.14231
		N	5	5	5	5	5	5
3	m	Mean	2.9650	0.08230	0.30860	5.4936	0.15214	0.57056
		S.D.	0.5888	0.02063	0.04753	1.2358	0.03906	0.09460
		N	5	5	5	5	5	5

Group	Sex		Ovaries Abs	Ovaries /BW	Ovaries /BR	Uterus/Cervix Abs	Uterus/Cervix /BW	Uterus/Cervix /BR
1	f	Mean	0.28754	0.00758	0.03178	7.4864	0.19694	0.82430
		S.D.	0.02775	0.00082	0.00307	1.1296	0.02486	0.08372
		N	5	5	5	5	5	5
2	f	Mean	0.38504	0.00910	0.04150	9.0394	0.20830	0.96896
		S.D.	0.08828	0.00224	0.00864	2.9930	0.04497	0.25999
		N	5	5	5	5	5	5
3	f	Mean	0.27454	0.00704	0.02812	5.2904	0.13568*	0.54020*
		S.D.	0.11358	0.00296	0.01149	1.0440	0.02721	0.08131
		N	5	5	5	5	5	5

* - Significantly different from the control value, $p \leq 0.05$

Nominal Dose: Group 1 – 0 µg Group 2 – 96 µg Group 3 - 96+171+527 µg

Absolute organ weights in grams (Abs), organ-to-body weight ratios (/BW), and organ-to-brain weight ratios (/BR)

Table S6D: Summary of Organ Weight Data

Day 85 Relative to Start Date

Group	Sex		Kidneys Abs	Kidneys /BW	Kidneys /BR	Liver/Gallbladder Abs	Liver/Gallbladder /BW	Liver/Gallbladder /BR	Pituitary Abs	Pituitary /BW	Pituitary /BR
1	m	Mean	17.1018	0.47032	1.76944	84.6974	2.31820	8.75304	0.03158	0.00084	0.00324
		S.D.	0.5803	0.03061	0.07390	8.2997	0.07869	0.75494	0.01287	0.00030	0.00131
		N	5	5	5	5	5	5	5	5	5
2	m	Mean	18.1380	0.46306	1.88166	101.0222	2.60214	10.50312	0.02882	0.00076	0.00298
		S.D.	3.2833	0.02996	0.33908	20.3916	0.50365	2.16749	0.01160	0.00032	0.00115
		N	5	5	5	5	5	5	5	5	5
3	m	Mean	16.8648	0.47168	1.73396	80.3812	2.24022	8.31160	0.03044	0.00084	0.00310
		S.D.	2.7461	0.06417	0.27012	16.7045	0.38808	1.87079	0.00768	0.00023	0.00062
		N	5	5	5	5	5	5	5	5	5
1	f	Mean	16.3984	0.37602	1.76762	79.0054	1.79892	8.52512	0.03412	0.00082	0.00364
		S.D.	1.4064	0.02032	0.23836	13.2254	0.15660	1.69786	0.01068	0.00028	0.00107
		N	5	5	5	5	5	5	5	5	5
2	f	Mean	16.3314	0.40140	1.71788	78.0462	1.91784	8.22614	0.04214	0.00104	0.00446
		S.D.	1.6990	0.04031	0.22479	7.3657	0.16178	1.13414	0.01023	0.00023	0.00133
		N	5	5	5	5	5	5	5	5	5
3	f	Mean	17.3664	0.39514	1.83324	83.2392	1.88210	8.81986	0.03542	0.00082	0.00374
		S.D.	2.7684	0.02630	0.26172	19.9827	0.26734	2.16366	0.00610	0.00019	0.00071
		N	5	5	5	5	5	5	5	5	5

Nominal Dose: Group 1 – 0 µg Group 2 – 96 µg Group 3 - 96+171+527 µg
Absolute organ weights in grams (Abs), organ-to-body weight ratios (/BW), and organ-to-brain weight ratios (/BR)

Table S6E: Summary of Organ Weight Data

Day 85 Relative to Start Date

Group	Sex		Spleen Abs	Spleen /BW	Spleen /BR	Thymus Abs	Thymus /BW	Thymus /BR	Thyroid/Parathyroid Abs	Thyroid/Parathyroid /BW	Thyroid/Parathyroid /BR
1	m	Mean	1.0490	0.02904	0.10878	4.0466	0.11048	0.41616	0.26042	0.00708	0.02688
		S.D.	0.2217	0.00769	0.02467	1.5620	0.04068	0.15324	0.08334	0.00219	0.00854
		N	5	5	5	5	5	5	5	5	5
2	m	Mean	1.3096	0.03376	0.13514	6.2874*	0.16280*	0.65488*	0.22886	0.00602	0.02376
		S.D.	0.4043	0.01105	0.03907	0.4695	0.02025	0.07747	0.05359	0.00177	0.00561
		N	5	5	5	5	5	5	5	5	5
3	m	Mean	1.1624	0.03260	0.11788	4.3622	0.12148	0.45316	0.21908	0.00602	0.02236
		S.D.	0.3934	0.01129	0.03333	1.2543	0.03280	0.14158	0.08746	0.00197	0.00815
		N	5	5	5	5	5	5	5	5	5
1	f	Mean	1.5134	0.03442	0.16206	7.1140	0.16400	0.76476	0.24912	0.00578	0.02684
		S.D.	0.4230	0.00766	0.04242	0.5592	0.01949	0.07974	0.05578	0.00151	0.00674
		N	5	5	5	5	5	5	5	5	5
2	f	Mean	1.4810	0.03660	0.15604	6.1254	0.14704	0.65238	0.24388	0.00594	0.02574
		S.D.	0.2638	0.00765	0.03181	2.4877	0.04784	0.29358	0.07340	0.00136	0.00862
		N	5	5	5	5	5	5	5	5	5
3	f	Mean	1.8420	0.04230	0.19518	6.3380	0.14284	0.67110	0.21918	0.00502	0.02310
		S.D.	0.1310	0.00364	0.01816	1.6922	0.02151	0.17749	0.05907	0.00134	0.00594
		N	5	5	5	5	5	5	5	5	5

* - Significantly different from the control value, $p \leq 0.05$

Nominal Dose: Group 1 – 0 µg Group 2 – 96 µg Group 3 - 96+171+527 µg
Absolute organ weights in grams (Abs), organ-to-body weight ratios (/BW), and organ-to-brain weight ratios (/BR)

Table S6F: Summary of Organ Weight Data

Day 85 Relative to Start Date

Group	Sex		Epididymides Abs	Epididymides /BW	Epididymides /BR	Testes Abs	Testes /BW	Testes /BR
1	m	Mean	3.0592	0.08394	0.31622	6.5298	0.17990	0.67790
		S.D.	0.2140	0.00422	0.01776	2.0128	0.05583	0.21819
		N	5	5	5	5	5	5
2	m	Mean	3.6488	0.09468	0.37864	6.4842	0.16770	0.67428
		S.D.	0.4224	0.01507	0.04069	0.2916	0.01502	0.05274
		N	5	5	5	5	5	5
3	m	Mean	2.8298	0.08018	0.28886	5.6458	0.16008	0.58122
		S.D.	0.6751	0.02391	0.05373	0.8709	0.03653	0.08820
		N	5	5	5	5	5	5

Group	Sex		Ovaries Abs	Ovaries /BW	Ovaries /BR	Uterus/Cervix Abs	Uterus/Cervix /BW	Uterus/Cervix /BR
1	f	Mean	0.41438	0.00946	0.04434	8.9228	0.20580	0.96240
		S.D.	0.10939	0.00235	0.01097	1.4707	0.04054	0.19543
		N	5	5	5	5	5	5
2	f	Mean	0.35964	0.00892	0.03732	8.5254	0.21592	0.87670
		S.D.	0.16248	0.00424	0.01631	3.7001	0.11214	0.30611
		N	5	5	5	5	5	5
3	f	Mean	0.48896	0.01116	0.05216	8.0436	0.18088	0.84826
		S.D.	0.18317	0.00402	0.02088	2.5236	0.03273	0.25029
		N	5	5	5	5	5	5

Nominal Dose: Group 1 – 0 µg Group 2 – 96 µg Group 3 - 96+171+527

Absolute organ weights in grams (Abs), organ-to-body weight ratios (/BW), and organ-to-brain weight ratios (/BR)
